# Effectiveness Evaluation of Levamisole, Albendazole, Ivermectin, and *Vernonia amygdalina* in West African Dwarf Goats

**DOI:** 10.1155/2015/706824

**Published:** 2015-10-22

**Authors:** Oyeduntan A. Adediran, Emmanuel C. Uwalaka

**Affiliations:** Department of Veterinary Microbiology and Parasitology, University of Ibadan, Ibadan, Oyo, Nigeria

## Abstract

Anthelmintic drug resistance has led to the search for alternatives in controlling helminth infections. Fifty West African Dwarf goats without history of anthelmintic treatment were divided equally into five groups. Group A was treated with ivermectin injection subcutaneously, group B with levamisole subcutaneously, group C with albendazole orally, and group D with aqueous extract of *Vernonia amygdalina* and group E was untreated control. Faecal samples were collected before treatment from each animal and larval culture was carried out. Faecal egg count reduction (FECR) test was carried out for each group and the data analysed using FECR version 4 to calculate percent reduction in faecal egg count. Predominant helminth infections from larval culture were *Haemonchus contortus* (70%), *Trichostrongylus* spp. (61%), and *Oesophagostomum* spp. (56%). Mixed infection was present in all the animals. From the FECR test *Vernonia amygdalina* extract was more effective against helminths (100%), compared to ivermectin 96%, levamisole 96%, and albendazole 99%. The lower 95% confidence limit was 89 for ivermectin and levamisole and 91 for albendazole. There is low resistance to ivermectin and levamisole and susceptibility to albendazole while *V. amygdalina* has great potentials that could be explored for the treatment of helminth diseases in goats.

## 1. Introduction

Gastrointestinal parasitic infection is one of the major health problems in the world. Huge economic losses have been seen in livestock production causing problems in animals and humans [1]. The use of chemotherapeutic drugs to control internal and external parasites is a widespread practice in livestock production and several broad-spectrum anthelmintics are available in the market for the control of helminthosis. Currently, failure of anthelmintics and reduced efficacy due to resistance of nematodes in sheep and goats are becoming a threat in some countries and are of increasing importance in certain African countries [2, 3]. According to Waller [4], most of the nematodes of domestic animals have shown resistance to common anthelmintics especially in warm and humid parts of the world and this has been suspected to be due to frequent dosing and poor therapeutic strategies [5–8].

Drug resistance was first reported in the late 1950's [9, 10] and early 1960's [11–14]. By the nineties, anthelmintic resistance had become a serious problem. Recently, lots of works have been done on this within and outside of Africa [15–22]. This has led to the search for a more eco-friendly anthelmintic that can tackle the resistance issue.* V. amygdalina* has been shown to have potentials as an anthelmintic in some animal species and humans [23–25]. Adediran et al. [23] worked on* V. amygdalina* as an anthelmintic on goats though it was the stalk and leaves that were fed raw to the goats. A comparison to establish the efficacy of this plant relative to well known and established synthetic anthelmintics to which resistance has been reported is therefore needed. We investigated the response of anthelmintic-naive goats that have acquired helminth infections naturally to treatment with three commonly used anthelmintics and* V. amygdalina* in southwest Nigeria.

Faecal egg count reduction test (FECRT), the commonest nematicide evaluation means [26, 27], has some shortcomings primarily due to the fact that egg count does not correspond to worm burdens. The FECRT can however be standardized by randomization of animals into treatment groups for even dispersion of egg counts and drug effectiveness, accurate dosing to make sure no administered drug is lost, and proper tagging of animals in each treatment group to ensure correct sampling. Our study was conducted with strict adherence to the standardisation method to ensure accuracy of our data on the drugs we evaluated.

The objective of this study was to evaluate resistance in West African Dwarf goats to levamisole, albendazole, and ivermectin and* V. amygdalina*.

## 2. Material and Method

### 2.1. Experimental Design

#### 2.1.1. Experimental Animals

Fifty goats were acquired from households without known history of anthelmintic treatment. The goat owners were interviewed before purchase to establish the treatment history of the herd. They were allowed to stabilize by giving feed, water, and mineral lick supplement. After two weeks, the animals were divided into four treatment groups (Group A, ivermectin treatment, Group B, levamisole treatment, Group C, albendazole treatment, and Group D,* V. amygdalina* treatment and Group E was untreated reference group).

### 2.2. Anthelmintic Treatment

The drugs (ivermectin, levamisole, and albendazole) were acquired from a veterinary drug store located in the study area while the plant,* V. amygdalina*, was collected from the environment and taken for authentication at the department of botany in the institution. The* V. amygdalina* leaves after identification were taken to the laboratory where the aqueous extract was prepared. Two kilograms of* V. amygdalina* leaves was hand-washed in two litres of distilled water and passed through a 2 mm sieve and the filtrate was used within one hour. Each animal in group A was given a dose of ivermectin injection of 0.2 mg/kg body weight subcutaneously individually. Each animal in group B was given levamisole injection at a dose rate of 8.0 mg/kg body weight subcutaneously; albendazole bolus was administered orally to each animal in group C at a dose rate of 10 mg/kg while the animals in group D were drenched with 5 mls per kg body weight of the plant aqueous extract. Group E was not given any form of anthelmintic treatment.

### 2.3. Faecal Sample Collection

Faecal samples were collected fresh from the rectum of the animals pretreatment and posttreatment in air-tight bags and taken to the laboratory for analysis. Posttreatment samples were collected on days 1, 2, 4, 7, and 14. The samples were then subjected to the faecal egg counts using the Mcmaster method described by [28]. The faecal culture, floatation, and sedimentation techniques of faecal examination were carried out as described by [29]. Faecal egg counts, helminth ova, and larvae identified were recorded.

### 2.4. Data Analysis

The percentage reduction in faecal egg count was calculated for each treatment group using the arithmetic mean of the egg count for each group at day 7 with the FECR version 4 software. Resistance to an anthelmintic was considered to be present if the percentage reduction in egg count was less than 95%, and the lower 95% confidence limit is less than 90.

## 3. Results

**Figure 1:**
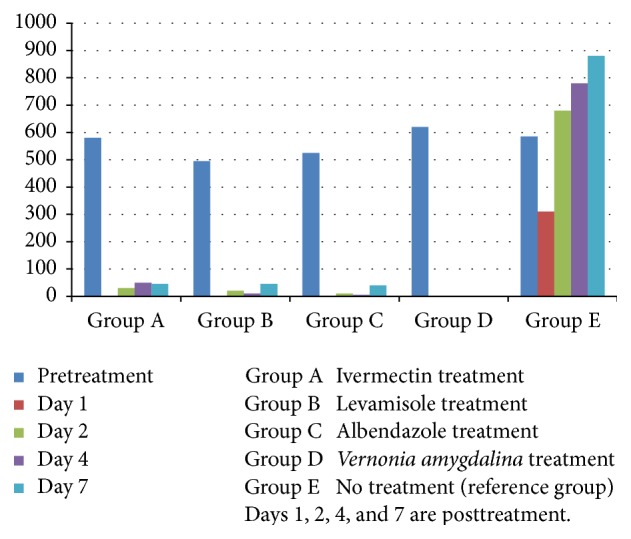
Graphical representation of the means for groups A–E.

The helminth ova identified during pretreatment faecal examination were* Haemonchus contortus*,* Trichostrongylus* spp.,* Ostertagia ostertagi*,* Oesophagostomum* spp.,* Chabertia *sp.,* Strongyloides*, and* Paramphistomum* spp. Larvae of* Haemonchus contortus*,* Trichostrongylus *spp.,* Ostertagia ostertagi*,* Oesophagostomum* spp.,* Charbetia* sp., and* Strongyloides* were identified at larva culture.* H. contortus* was identified in 70% of the goats,* Trichostrongylus* in 61%,* Oesophagostomum* in 56%,* Chabertia* in 15%, and* Strongyloides* in 10%. Posttreatment,* H. contortus* larvae were the most common followed by* Trichostrongylus* spp. and* Oesophagostomum* spp. The percentage reduction in faecal egg count was 96% for each of ivermectin, levamisole, and albendazole and 100% for* V. amygdalina*. The statistical analysis showed a lower 95% confidence limit (CL) of 89 for ivermectin and levamisole and 91 for albendazole (Table 1). This result implies that there is low resistance in nematodes of goats to ivermectin and levamisole while there is susceptibility to albendazole and* V. amygdalina*. The arithmetic means of the faecal egg count for the different groups are presented in Figure 1.

## 4. Discussion

Anthelmintic drugs such as albendazole, ivermectin, and levamisole are used to treat parasitic infection in ruminants. The regular use of these drugs and sometimes improper use make the issue of resistance inevitable. According to [30], it has been observed that frequent use of the same group of anthelmintic, use of anthelmintics in suboptimal doses, prophylactic mass treatment of domestic animals, and frequent and continuous use of a single drug have contributed to the widespread development of anthelmintic resistance in helminths. Research reports of anthelmintic resistance in goats in Africa have been relatively few although this is more likely to be as a result of low number of studies carried out in this area and not because cases of resistance were not observed. This present study showed that there is a measure of resistance to ivermectin and levamisole in Nigerian WAD goats while albendazole is still an effective drug. Also* V. amygdalina* has been shown to be an alternative source of treating helminths in goats. It is recommended to keep animals on dry lot for 12 to 24 hours after deworming to ensure that eggs and larvae that survive the anthelmintics are not deposited on safe pasture [31]. This presupposes that 24 hours after treatment there should not be viable helminth eggs; therefore, the hatching of viable eggs seven days after treatment in our study is an indication that the worms survived. Ivermectin, being the most widely used because of its effect against ectoparasites, has been grossly overused; hence it is highly susceptible to resistance development. This is corroborated by the works of [20] who worked in Howell et al. [18] in the United States. Our results however differ from the works of [15] who worked on ivermectin in sheep nematodes in Oyo State, Nigeria, but did not record any resistance. This is possibly as a result of the different assays used and the species of small ruminant involved. Ademola [15] used sheep and the Larval Development Assay (LDA) while goats were used in this study and the FECRT was used as the assay. Although goats and sheep have similar genera of nematodes, it has been reported that nematodes in goats herd usually develop anthelmintic resistance more rapidly [32]. Also, the FECRT we used is an* in vivo* assay while LDA is an* in vitro* assay which according to [33] may be useful as a supplement to FECRT. In Ethiopia, the reports [34–39] that albendazole, ivermectin, and levamisole were effective in goats though resistance to albendazole was suspected are also at variance with the results in this study. Difference in location, brand of drug available, and breed of goat used for experiments and level of resistance already developed by helminths are likely reasons for the disparity. Levamisole in goats has been known to produce adverse reactions such as muscle tremors and hyperesthesia with irritability even at recommended doses; subsequently the subcutaneous administration inducing a lower blood peak of the drug is often preferred in goats [40]. Based on the adverse reactions in goats, levamisole is therefore often administered with caution using the lower dose range which may at times result in suboptimal dosing when given through the subcutaneous route, resulting in the gradual development of resistance. The 100% efficacy recorded for* V. amygdalina* is also corroborated by an earlier report by [23]. The result also authenticates the reports of [8, 25, 41]. The implication of this is that the plant could be a good alternative to the commonly used synthetic anthelmintics. The plant is also readily available and it is found as a shrub in most rural backyard gardens in developing countries to which Nigeria belongs [42].

## 5. Conclusion

The study has shown that the common anthelmintics used in West African Dwarf (WAD) goats in Nigeria are still effective although low level of resistance was recorded for ivermectin and levamisole. However, the presence of any degree of resistance is of concern to small ruminant production. Also, we conclude that* Vernonia amygdalina* presents a ready alternative that would likely take care of anthelmintic resistance in common goat nematodes.

Strategies recommended to control helminths include a better use of existing drugs, use of vaccines for helminths, growth regulators, and biological control [5]. However, maintaining parasites in refugia and not exposing them to anthelmintics have been identified as key in controlling and delaying the development of resistance because susceptible genes are preserved [30]. According to Chiejina and Behnke [43], the WAD goats should be explored for their unique resilience and genetic improvement and looked into reducing the use of chemical anthelmintics. In line with these recommendations, better drug-use and control as well as targeted and planned treatment routines should be carried out to keep resistance low and ensure adequate control of helminth parasites in WAD in southwest Nigeria. Use of ethnoveterinary plants like* V. amygdalina* as an alternative source for effective anthelmintic treatment is also highly recommended. The plant presents an environmentally friendly alternative that will essentially reduce reliance on use of chemicals.

## Figures and Tables

**Table 1:** Faecal egg count reduction.

	Reference (control)	Ivermectin	Levamisole	Albendazole	*V*. *amygdalina*
Number	8	10	10	10	10
Arithmetic mean	1100	45	45	40	0
Variance (FEC)	310000	3583	3583	2667	0
% reduction	—	96	96	96	100
Variance (reduction)	—	0.21	0.21	0.20	—
Upper 95% CL	—	98	98	99	—
Lower 95% CL	—	89	89	91	—
